# An Efficient Method for the Preparative Isolation and Purification of Flavonoids from Leaves of *Crataegus pinnatifida* by HSCCC and Pre-HPLC

**DOI:** 10.3390/molecules22050767

**Published:** 2017-05-09

**Authors:** Lei Wen, Yunliang Lin, Ruimin Lv, Huijiao Yan, Jinqian Yu, Hengqiang Zhao, Xiao Wang, Daijie Wang

**Affiliations:** 1 College of Pharmacy, Shandong University of Traditional Chinese Medicine, 4655 Daxue Road, Jinan 250355, China; 18363081657@163.com; 2 Shandong Key Laboratory of TCM Quality Control Technology, Shandong Analysis and Test Center, Shandong Academy of Sciences, 19 Keyuan Street, Jinan 250014, China; wodekafei@126.com (Y.L.); lvrm@sdatc.com.cn (R.L.); yanhuijiao01@163.com (H.Y.); yujinqian87528@126.com (J.Y.); hqzhao2007@163.com (H.Z.); wxjn1998@126.com (X.W.)

**Keywords:** *Crataegus pinnatifida* leaves, flavonoids, HSCCC and pre-HPLC combination, extrusion mode

## Abstract

In this work, flavonoid fraction from the leaves of *Crataegus pinnatifida* was separated into its seven main constituents using a combination of HSCCC coupled with pre-HPLC. In the first step, the total flavonoid extract was subjected to HSCCC with a two-solvent system of chloroform/methanol/water/*n*-butanol (4:3:2:1.5, *v/v*), yielding four pure compounds, namely (–)-epicatechin (**1**), quercetin-3-*O*-(2,6-di-α-l-rhamnopyranosyl)-β-d-galactopyranoside (**2**), 4′′-*O*-glucosylvitexin (**3**) and 2′′-*O*-rhamnosylvitexin (**4**) as well as a mixture of three further flavonoids. An extrusion mode was used to rapidly separate quercetin-3-*O*-(2,6-di-α-l-rhamnopyranosyl)-β-d-galactopyranoside with a big *K*_D_-value. In the second step, the mixture that resulted from HSCCC was separated by pre-HPLC, resulting in three pure compounds including: vitexin (**5**), hyperoside (**6**) and isoquercitrin (**7**). The purities of the isolated compounds were established to be over 98%, as determined by HPLC. The structures of these seven flavonoids were elucidated by ESI-MS and NMR spectroscopic analyses.

## 1. Introduction

*Crataegus pinnatifida*, Rosaceae family, is widely spread in Northern China and is considered as a famous medicinal and edible plant. The leaves of *C. pinnatifida*, a famous folk medicine, have been used clinically for dredging the meridian, lowering blood lipid levels, activating blood flow and removing blood stasis [1]. So far, many compounds including flavonoids, triterpenoids, steroids, monoterpenoids, sesquiterpenoids, lignans, hydroxycinnamic acids, organic acids and nitrogen-containing compounds have been isolated and identified from *C. pinnatifida*. Among them, the flavonoids are considered to be the major bioactive constituents with many pharmacological actions, such as treating fatty livers, showing antioxidant activity, and exhibiting a protective effect on cell toxicity and protease inhibitory activities [2,3,4,5,6].

Previously, flavonoids from the leaves of *C. pinnatifida* were isolated and purified by conventional techniques, such as polyamide chromatography, silica gel column chromatography and sephadex LH-20, which were a waste organic solvent, tedious, time-consuming and often resulted in irreversible adsorption of the compounds [7,8,9,10,11,12,13,14]. Thus, effective separation methods for flavonoids from the leaves of *C. pinnatifida* are of great importance.

High-speed counter-current chromatography (HSCCC) has now been widely applied in the separation of various compounds, as a liquid-liquid partition chromatography [15]. Generally speaking, HSCCC needs no solid support, which indicates that it possesses high loading capacity, low economic cost and no irreversible adsorption of samples. Therefore, HSCCC has been developed to be an efficient tool for the purification and separation of various samples [16,17,18,19,20,21,22].

**Figure 1:**
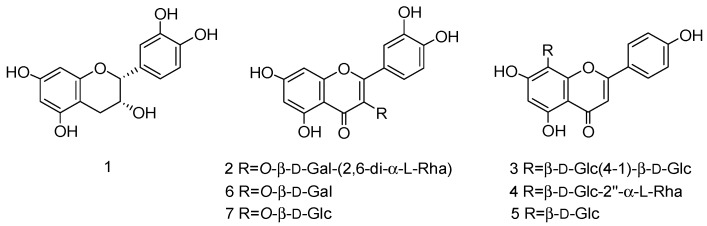
Structures of the compounds from *C. pinnatifida.*

In a previous study, three flavonoids from the leaves of *C. pinnatifida* were isolated using HSCCC coupled with macroporous resin and pre-HPLC [23]. In that work, the solvent system was selected by analytical HSCCC, and did not measure the *K*_D_-values. Furthermore, the flavonoids from the leaves of *C. pinnatifida* were partial, acquired by 20% ethanol elution of D101 macroporous resin. In this work, a new two-phase solvent system of HSCCC was developed for the purification and separation of flavonoids from the leaves of *C. pinnatifida*. Furthermore, an extrusion mode was used to rapidly separate compounds with big *K*_D_-values. The mixture was further purified by pre-HPLC. Finally, seven flavonoids were separated and elucidated by ESI-MS and NMR spectroscopic analyses. The structures of these compounds are shown in Figure 1.

## 2. Results and Discussion

### 2.1. Optimization of HSCCC Conditions

The separation of the seven flavonoids from the leaves of *C. pinnatifida* was carried out by HSCCC. A successful separation of the target compounds using HSCCC required a careful search for a suitable two-phase solvent system to provide an ideal range of *K*_D_-values. According to the structural characteristics of flavonoids, three series of solvent systems including *n*-hexane/ethyl acetate/methanol/water, *n*-hexane/ethyl acetate/ethanol/water and chloroform/ethanol/water were designed on the basis of previous research [24]. The *K*_D_-values of the target compounds in the three series of solvent systems were detected and calculated, as shown in Table 1.

When *n*-hexane/ethyl acetate/methanol/water (1:5:1:5, *v/v*) was used (the lower phase as the mobile), the *K*_D_-values of the compounds **2**–**7** were too small, resulting in a poor resolution. Using *n*-hexane/ethyl acetate/ethanol/water (1:1.6:1:1.6, *v/v*) did not result in any significant change. Therefore, the above two series of solvent systems were not fit to separate the flavonoids from the leaves of *C. pinnatifida*. Chloroform/ethanol/water (4:3:2, *v/v*) could provide a wide range of *K*_D_-values. As the *n*-butanol ratio was added and increased, the *K*_D_-values of the target compounds could decrease. When the ratio was up to chloroform/methanol/water/*n*-butanol (4:3:2:2, *v/v*), the solution could not be layered. Considering these comprehensive factors, chloroform/methanol/water/*n*-butanol (4:3:2:1.5, *v/v*) was chosen. However, the *K*_D_-value of compound **2** was too large. If a conventional elution was used, the separation time of compound 2 was too long, resulting in a waste of solvent. Hence, an extrusion mode was used to rapidly separate compound **2** by effluent collected in tubes after the HSCCC equipment stopped with a head-to-tail mode.

**Figure 2:**
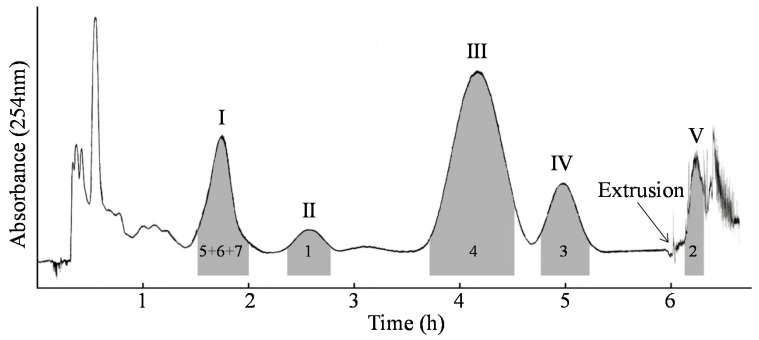
Chromatogram of the purchased total flavonoids from the leaves of *C. pinnatifida* by HSCCC. Solvent system: chloroform/methanol/water/*n*-butanol (4:3:2:1.5, *v/v*); Mobile phase: the lower; Revolution speed: 850 rpm; Flow rate: 5.0 mL/min; Sample size: 200 mg; Injection volume: 20 mL; Wavelength: 254 nm; Stationary phase retention: 62.8%.

The purchased total flavonoids from the leaves of *C. pinnatifida* were first purified under chloroform/methanol/water/*n*-butanol (4:3:2:1.5, *v/v*). The total separation time of HSCCC was about 7 h with the stationary phase of 62.8%. Based on HPLC analysis of Figure 2, four pure compounds of peak II (**1**, 8.9 mg), peak III (**4**, 47.3 mg), peak IV (**3**, 18.2 mg), and peak V (**2**, 10.3 mg) were obtained, with purities over 98%, respectively, as determined by HPLC. Also, a mixture (25.9 mg) of peak I was obtained which was composed of **5**–**7**.

In a previous study, a two-solvent system composed of *n*-butanol–water (1:1, *v/v*) was used to separate flavonoids from the leaves of *C. pinnatifida*. The stationary phase retention of *n*-butanol–water (1:1, *v/v*) was 40%. A pure compound of 2′′-*O*-rhamnosylvitexin and a mixture were obtained in a one-step HSCCC separation. In the present study, the stationary phase retention of the selected chloroform/methanol/water/*n*-butanol (4:3:2:1.5, *v/v*) was 62.8%. The increase of the stationary phase retention indicates that the number of theoretical plates and the separation efficiency increased. In addition, four pure compounds and a mixture were yielded in a one-step HSCCC separation. This developed solvent system and methodology could be applied to rapidly separate pure compounds in further research on *C. pinnatifida* and its flavonoids.

### 2.2. Pre-HPLC Separation

**Figure 3:**
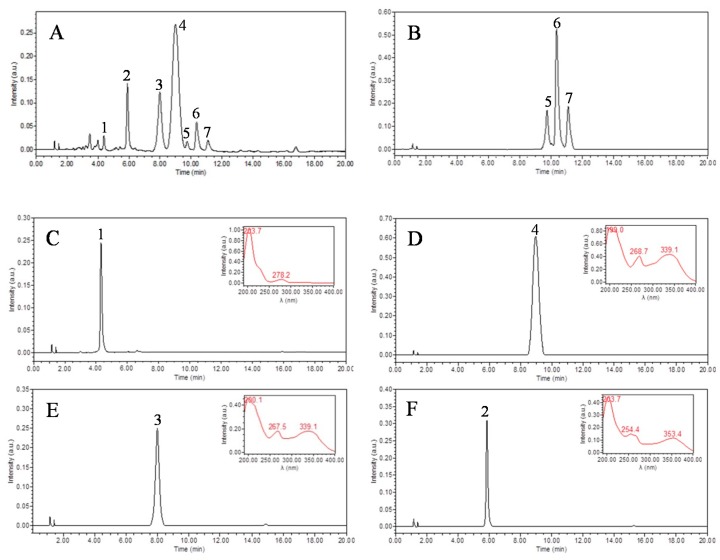
HPLC chromatograms of HSCCC fractions. (**A**: purchased total flavonoids; **B**: peak I in Figure 2; **C**: peak II in Figure 2; **D**: peak III in Figure 2; **E**: peak IV in Figure 2; **F**: peak V in Figure 2.) Experimental conditions: a Waters XBridge BEH C_18_ column (100 mm × 4.6 mm i.d., 2.5 μm); Flow rate: 1.0 mL/min; Column temperature: 25 °C; Injection volume: 10 μL; Detection: 254 nm. HPLC conditions are as follows: acetonitrile (**A**) and water (**B**), the gradient elution mode was set as follows: 0–3 min, 13–14% A; 3–15 min, 14–17% A; 15–15.1 min, 17–13% A; 15.1–20 min, 13% A.

**Figure 4:**
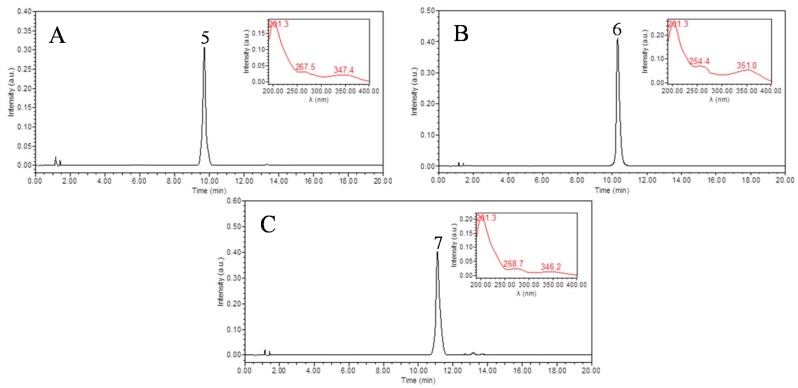
Chromatograms of pre-HPLC fractions. (**A**: vitexin, **5** in Figure 3A; **B**: hyperoside, **6** in Figure 3A; **C**: isoquercitrin, **7** in Figure 3A).

The peaks I-V isolated by HSCCC were analyzed by HPLC (Figure 3). Peak I containing compounds **5**–**7** were further purified by pre-HPLC with a solvent of acetonitrile–water (19:81, *v/v*). The flow-rate was set at 3.0 mL/min with a wavelength of 254 nm. Finally, three compounds were obtained with 4.6 mg of vitexin, 11.7 mg of hyperoside, and 9.2 mg of isoquercitrin, with the purities over 98%, respectively, as determined by HPLC (Figure 4).

## 3. Materials and Method

### 3.1. Apparatus and Materials

HSCCC separation was conducted on a TBE-300C (Tauto Biotechnique, Shanghai, China), which was equipped with a 300-mL PTFE multilayer coil (diameter of the PTFE tube as 1.9 mm) as well as a 20-mL manual sample loop. The rotation speed of the column coil could be adjustable from 0 to 1000 rpm. The HSCCC apparatus was also equipped with four other instrument modules, including a TBP-5002 constant-flow pump (Tauto Biotechnique, Shanghai, China), a 8823A-UV Monitor at 254 nm (Beijing Emilion Technology, Beijing, China), a Model 3057 portable recorder (Yokogawa, Sichuan Instrument Factory, Sichuan, China) and a DC-0506 low constant temperature bath (Tauto Biotechnique, Shanghai, China) to maintain the temperature at 25 °C.

A Waters e2695 equipment with a 2695 quaternary-solvent delivery system, a 2998 Photodiode Array Detection (DAD) detector, an automatic sample injection, a 2695 column oven and an Empower 3 ChemStation was used to analyze the crude extract and the collected fractions. The column used was a Waters XBridge BEH C_18_ column (250 mm × 4.6 mm, i.d., 5 μm, Waters, Milford, MA, USA).

*n*-Hexane, ethyl acetate, *n*-butanol, chloroform and ethanol used for separation were all of analytical grade (Sinopharm Chemical Reagent Co., Ltd., Shanghai, China). The acetonitrile of HPLC grade used in HPLC analysis was purchased from Fisher Scientific (Fair Lawn, NJ, USA). The water used was deionized by an osmosis Milli-Q system (Millipore, Bedford, MA, USA).

The raw material of purified flavonoid fraction from the leaves of *C. pinnatifida* was purchased from Shannxi Haochen Biotechnology Co., Ltd., Chengdu, China. The contents of flavonoids were approximately 80%, respectively, as determined by HPLC.

### 3.2. Selection of the Two-Phase Solvent System

2 mg of total flavonoids extract was dissolved in 2 mL lower phase and detected by HPLC, recorded as *A*_1_ of the peak area. Then, 2 mL upper phase was added to the solution and mixed thoroughly. After 3 minutes standing, the lower was detected by HPLC, recorded as *A*_2_. The *K*_D_-value was calculated by the following equation: *K*_D_ = (*A*_1_ − *A*_2_)/*A*_2_ [25].

### 3.3. Preparation of the Two-Phase Solvent System and Sample Solution

The solvent system of chloroform/methanol/water/*n*-butanol (4:3:2:1.5, *v/v*) was selected as the optimum system in the HSCCC experiment. After thoroughly equilibrating in a separation funnel, the solvent system was divided into two separated phases before use. The upper phase was used as the stationary phase, while the lower was used as the mobile phase.

200 mg of raw flavonoid sample was dissolved in 20 mL of mobile phase and stationary phase (1:1) as a sample solution.

### 3.4. HSCCC Separation Procedure

For the HSCCC experiment, the separation column was initiated by being filled with the upper phase at 30.0 mL/min, and then the column was rotated at 850 rpm. The flow-rate of mobile phase was 5.0 mL/min. After the equilibration was reached, the sample solution was injected into the sample loop. Fractions were manually collected by HSCCC chromatogram. The retention of the stationary phase retention was defined as the stationary phase relative to the total column capacity after separation.

### 3.5. HPLC analyses of HSCCC and Pre-HPLC Peak Fractions

The total flavonoids and each peak fraction from HSCCC separation were analyzed by HPLC. The mobile phase was acetonitrile (A) and water (B); the gradient elution mode was set as follows: 0–3 min, 13–14% A; 3–15 min, 14–17% A; 15–15.1 min, 17–13% A; 15.1–20 min, 13% A with a flow-rate of 1.0 mL/min.

Pre-HPLC separations were used with a YMC C_18_ column (10.0 mm × 250 mm, 5 μm) with a solvent of acetonitrile–water (19:81, *v/v*) at a flow-rate of 3.0 mL/min, and monitored at 254 nm.

### 3.6. Electrospray Ionization Mass Spectrometry (ESI-MS) and Nuclear Magnetic Resonance Spectroscopy (NMR)

ESI-MS experiments were performed on an Agilent 6520 Q-TOF (Agilent, Santa Clara, CA, USA) and NMR spectra were performed on a Bruker AV-400 spectrometer (Bruker BioSpin, Rheinstetten, Germany) with TMS as an internal standard.

### 3.7. Identification of the Isolated Compounds

Seven flavonoid compounds were isolated. Their structures were identified by comparison of their spectroscopic data reported, including ESI-MS and NMR data. 

*(−)-Epicatechin* [26] (**1**, Figure 3C): ESI-MS, *m*/*z* 289.1 [M − H]^−^, 579.2 [2M − H]−. 1H-NMR (400 MHz, DMSO-*d*_6_) *δ*: 9.11 (1H, br s, OH), 8.82–8.89 (3H, br s, OH), 6.90 (1H, d, *J* = 1.2 Hz, H-2′), 6.68 (1H, d, *J* = 8.0 Hz, H-5′), 6.65 (1H, dd, *J* = 8.0 Hz, 1.2Hz, H-6′), 5.89 (1H, d, *J* = 2.4 Hz, H-6), 5.70 (1H, d, *J* = 2.4 Hz, H-8), 4.74 (1H, s, H-2), 4.00 (1H, br s, H-3), 2.68 (1H, dd, *J* = 4.4, 16.4 Hz, H-4ax), 2.47 (1H, dd, *J* = 3.2, 16.4 Hz, H-4eq). ^13^C-NMR (100 MHz, DMSO-*d*_6_) *δ*: 157.0 (C-7), 156.7 (C-9), 156.2 (C-5), 145.0 (C-3′), 144.9 (C-4′), 131.1 (C-1′), 118.4 (C-4′), 115.4 (C-2′), 115.3 (C-5′), 99.0 (C-10), 95.6 (C-6), 94.6 (C-8), 78.5 (C-2), 65.4 (C-3), 28.7 (C-4).

*Quercetin-3-O-(2,6-di-*α*-L-rhamnopyranosyl)-*β*-d-galactopyranoside* [27,28] (**2**, Figure 3F): ESI-MS, *m*/*z* 757.3 [M + H]^+^. ^1^H-NMR (400 MHz, DMSO-*d*_6_) *δ*: 7.69 (1H, dd, *J* = 2.0, 8.4 Hz, H-6′), 7.49 (1H, d, *J* = 2.0 Hz, H-2′), 6.82 (1H, d, *J* = 8.4 Hz, H-5′), 6.40 (1H, br s, H-8), 6.20 (1H, br s, H-6), 5.58 (1H, d, *J* = 7.6 Hz, gal H-1), 5.06 (1H, s, rha-a H-1), 4.39 (1H, s, rha-b H-1), 1.05 (3H, d, *J* = 6.0 Hz, rha-b H-6), 0.80 (3H, d, *J* = 6.0 Hz, rha-a H-6). ^13^C-NMR (100 MHz, DMSO-*d*_6_) *δ*: 177.7 (C-4), 164.6 (C-7), 161.7 (C-5), 156.7 (C-2), 156.7 (C-9), 148.8 (C-4′), 145.3 (C-3′), 133.3 (C-3), 122.5 (C-6′), 121.6 (C-1′), 116.2 (C-2′), 115.7 (C-5′), 104.4 (C-10), 101.0 (rha-a C-1), 100.5 (rha-b C-1), 99.5 (gal C-1), 99.2 (C-6), 94.0 (C-8), 75.3 (gal C-2), 74.4 (gal C-3), 73.8 (gal C-5), 72.4 (rha-a C-4), 72.4 (rha-b C-4), 71.9 (rha-a C-3), 71.1 (rha-a C-2), 71.1 (rha-b C-2), 70.9 (rha-b C-3), 69.0 (gal C-4), 68.7 (rha-a C-5), 68.6 (rha-b C-5), 65.5 (gal C-6), 18.4 (rha-b C-6), 17.7 (rha-a C-6).

*4′′-O-Glucosylvitexin* [23] (**3**, Figure 3E): ESI-MS, *m*/*z* 593.2 [M − H]^−^. ^1^H-NMR (400 MHz, DMSO-*d*_6_) *δ*: 13.15 (1H, s, 5-OH), 8.01 (2H, d, *J* = 8.8 Hz, H-2′, 6′), 6.90 (2H, d, *J* = 8.8 Hz, H-3′, 5′), 6.73 (1H, s, H-3), 6.24 (1H, s, H-6), 4.86 (1H, d, *J* = 10.0 Hz, H-1′′′), 4.80 (1H, d, *J* = 10.0 Hz, H-1′′). ^13^C-NMR (100 MHz, DMSO-*d*_6_) *δ*: 182.4 (C-4), 164.2 (C-2), 162.9 (C-9), 161.2 (C-4′), 160.4 (C-7), 156.7 (C-5), 129.3 (C-6′), 129.1 (C-2′), 122.2 (C-1′), 116.6 (C-5′), 116.3 (C-3′), 105.6 (C-3), 104.2 (C-10), 104.0 (C-8), 103.1 (C-1′′′), 98.7 (C-6), 82.2 (C-1′′), 81.6 (C-4′′), 78.9 (C-5′′), 76.7 (C-5′′′), 76.5 (C-3′′′), 74.8 (C-2′′′), 72.0 (C-3′′), 70.5 (C-2′′), 70.4 (C-4′′′), 61.4 (C-6′′), 60.9 (C-6′′′).

*2′′-O-Rhamnosylvitexin* [29] (**4**, Figure 4C): ESI-MS, *m*/*z* 579.2 [M + H]^−^, 577.2 [M − H]^−^. ^1^H-NMR (400 MHz, DMSO-*d*_6_) *δ*: 13.15 (1H, s, 5-OH), 8.05 (2H, d, *J* = 8.8 Hz, H-2′, 6′), 6.91 (2H, d, *J* = 8.8 Hz, H-3′, 5′), 6.79 (1H, s, H-3), 6.28 (1H, s, H-6), 4.98 (1H, s, H-1′′′), 4.77 (1H, d, *J* = 10.0 Hz, H-1′′), 0.48 (3H, d, *J* = 6.0 Hz, H-6′′′). ^13^C-NMR (100 MHz, DMSO-*d*_6_) *δ*: 182.5 (C-4), 164.4 (C-2), 162.7 (C-7), 161.5 (C-5), 161.1 (C-4′), 156.3 (C-9), 129.4 (C-6′), 129.1 (C-2′), 122.1 (C-1′), 116.5 (C-5′), 116.3 (C-3′), 104.9 (C-10), 104.7 (C-8), 103.0 (C-3), 100.8 (C-l′′′), 98.7 (C-6), 82.2 (C-5′′), 80.2 (C-3′′), 75.5 (C-2′′), 72.1 (C-1′′), 71.8 (C-4′′′), 71.0 (C-2′′′), 70.8 (C-3′′′), 70.6 (C-4′′), 68.7 (C-5′′′), 61.5 (C-6′′), 18.2 (C-6′′′).

*Vitexin* [30] (**5**, Figure 4A): ESI-MS, *m/z* 433.3 [M + H]^+^, 431.1 [M − H]^−^. ^1^H-NMR (400 MHz, DMSO-*d*_6_) *δ*: 13.16( 1H, s, 5-OH), 6.90 (2H, d, *J* = 6.4 Hz, H-3′, 5′), 6.77(1H, s, H-3), 6.26 (1H, s, H-6), 4.69 (1H, d, *J* = 9.9 Hz, H-1′′). ^13^C-NMR (100 MHz, DMSO-*d*_6_) *δ*: 181.7 (C-4), 163.7 (C-2), 162.7 (C-7), 161.0 (C-5), 160.3 (C-4′), 156.5 (C-9), 129.6 (C-2′), 128.8 (C-6′), 121.5 (C-1′), 115.7 (C-3′), 115.7 (C-5′), 104.7 (C-8), 104.1 (C-10), 102.4 (C-3), 98.2 (C-6), 81.7 (C-5′′), 78.5 (C-3′′), 73.3 (C-1′′), 70.8 (C-2′′), 70.4 (C-4′′), 61.1 (C-6′′).

*Hyperoside* [31] (**6**, Figure 4B): ESI-MS, *m*/*z* 465.2 [M + H]^−^, 463.1 [M − H]^−^. ^1^H-NMR (400 MHz, DMSO-*d*_6_) *δ*: 12.64 (1H, s, 5-OH), 10.84 (1H, s, 7-OH), 7.67 (1H, dd, *J* = 2.0, 8.4 Hz, H-6′), 7.54 (1H, d, *J* = 2.0 Hz, H-2′), 6.82 (1H, d, *J* = 8.4 Hz, H-6′), 6.41 (1H, d, *J* = 1.6 Hz, H-8), 6.20 (1H, d, *J* = 1.6 Hz, H-6), 5.38 (1H, d, *J* = 8.0 Hz, H-1′′). ^13^C-NMR (100 MHz, DMSO-*d*_6_) *δ*: 177.3 (C-4), 164.5 (C-7), 161.1 (C-5), 156.2 (C-9), 156.1 (C-2), 148.4 (C-4′), 144.7 (C-3′), 133.4 (C-3), 121.9 (C-6′), 121.0 (C-1′), 115.8 (C-5′), 115.1 (C-2′), 103.7 (C-10), 101.8 (C-1′′), 98.7 (C-6), 93.5 (C-8), 75.7 (C-5′′), 73.1 (C-3′′), 71.1 (C-2′′), 67.8 (C-4′′), 60.0 (C-6′′).

*Isoquercitrin* [32] (**7**, Figure 3D): ESI-MS, *m*/*z* 463.2 [M − H]^−^. ^1^H-NMR (400 MHz, DMSO-*d*_6_) *δ*: 12.75 (1H, s, 4′-OH), 7.58 (1H, dd, *J* = 6.0, 2.0 Hz, H-6′), 7.57 (1H, d, *J* = 2.0 Hz, H-2′), 6.89 (1H, d, *J* = 8.4 Hz,, H-5′), 6.41 (1H, d, *J* = 2.0 Hz, H-8), 6.21 (1H, d, *J* = 2.0 Hz, H-6), 5.46 (1H, d, *J* = 7.2 Hz, H-1′′). ^13^C-NMR (100 MHz, DMSO-*d*_6_) *δ*: 177.8 (C-4), 164.7 (C-7), 161.7 (C-5), 156.8 (C-9), 156.5 (C-2), 148.9 (C-4′), 145.3 (C-3′), 133.7 (C-3), 122.0 (C-6′), 121.6 (C-1′), 116.6 (C-5′), 115.7 (C-2′), 103.7 (C-10), 101.4 (C-1′′), 99.2 (C-6), 94.0 (C-8), 77.9 (C-5′′), 76.8 (C-3′′), 74.5 (C-2′′), 70.3 (C-4′′), 61.3 (C-6′′).

## 4. Conclusions

**Figure 5:**
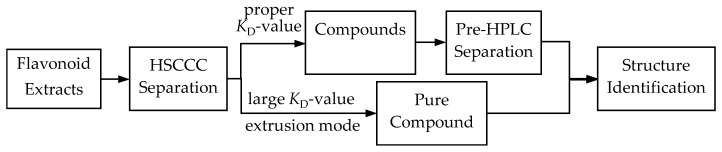
Diagram of the article procedure.

In the present study, an efficient method combining HSCCC and pre-HPLC was used to preparative separate flavonoids from the leaves of *C. pinnatifida* (Figure 5). In HSCCC separation, a solvent system of chloroform/methanol/water/*n*-butanol (4:3:2:1.5, *v/v*) was used to isolate the total flavonoids. *n*-Butanol was used to adjust the *K*_D_-values. For the compound with the largest *K*_D_-value, an extrusion mode was used ensure rapid separation. Pre-HPLC was then applied to separate compounds with close *K*_D_-values. Finally, seven compounds with high purities were obtained with the established method. The proposed method proved to be efficient to separate compounds with broad *K*_D_-values and similar polarities. This developed methodology could be applied to rapidly separate pure compounds in further research on *C. pinnatifida* and its flavonoids. It also could be used to obtain compounds for biological studies.

## Figures and Tables

**Table 1:** The K*_D_*-values of flavonoids from the leaves of *C. pinnatifida.*

Solvent System	*K*_D_-Values						
	1	2	3	4	5	6	7
*n*-Hex–EtOAc–MeOH–H_2_O (1:5:1:5, *v/v*)	4.43	0.40	0.41	0.12	<0.1	0.34	0.21
*n*-Hex–EtOAc–EtOH–H_2_O (1:1.6:1:1.6, *v/v*)	0.12	<0.1	<0.1	<0.1	<0.1	0.19	0.22
CHCl_3_–MeOH–H_2_O (4:3:2, *v/v*)	7.62	>30	13.95	10.43	4.39	4.05	4.11
CHCl_3_–MeOH–H_2_O–*n*-BuOH (4:3:2:0.5, *v/v*)	5.13	>30	12.25	8.04	3.46	3.02	3.12
CHCl_3_–MeOH–H_2_O–*n*-BuOH (4:3:2:1, *v/v*)	3.88	26.49	8.30	7.68	3.25	2.91	2.99
CHCl_3_–MeOH–H_2_O–*n*-BuOH (4:3:2:1.5, *v/v*)	2.15	18.91	4.78	3.35	1.47	1.33	1.34
